# Extracellular matrix-derived mechanical force induces CDK4/6 inhibitor resistance by inhibiting NEK10 dependent cell cycle regulation in breast cancer

**DOI:** 10.1097/JS9.0000000000004527

**Published:** 2026-01-12

**Authors:** Cong Li, Jian Wang, Yuming Jin, Yumeng Huang, Ruoxi Hong, Dongshao Chen, Shaoyan Lin, Limin Chen, Huailiang Wu, Ting Du, Binghe Xu, Wan Wang, Shusen Wang

**Affiliations:** a Department of Medical Oncology, State Key Laboratory of Oncology in South China, Guangdong Provincial Clinical Research Center for Cancer, Sun Yat-sen University Cancer Center, Guangdong, China; b Department of Oncology, Medical School of Tianjin University, Tianjin, China; c Department of General Surgery, Tianjin Fifth Central Horpital, Tianjin, China; d Department of Urology, Institute of Urology and National Clinical Research Center for Geriatrics, West China Hospital of Sichuan University, Chengdu, China; e Neurology Department, University of Texas Medical Branch, Galveston, Texas, the United States; f Non-coding RNA and Drug Discovery Key Laboratory of Sichuan Province, Department of Immunology, School of Basic Medical Sciences, Chengdu Medical College, Chengdu, China; g Department of Medical Oncology, National Cancer Center/National Clinical Research Center for Cancer/Cancer Hospital, Chinese Academy of Medical Sciences and Peking Union Medical College, Beijing, China; h Department of Breast Surgery, China-Japan Union Hospital of Jilin University, Jilin, China

**Keywords:** biomechanical force, CDK4/6 inhibitor, cell cycle, extracellular matrix, NEK10

## Abstract

**Background:**

Biomechanical signals play a pivotal role in tumor initiation and progression, with the extracellular matrix (ECM) acting as a key source of these signals. This study aims to investigate the role of ECM-derived biomechanical signals in mediating CDK4/6 inhibitor resistance in HR+, HER2− breast cancer.

**Materials and methods:**

This study utilized 3D Matrigel, collagen, and fibrin gels to examine the role of ECM-derived biomechanical signals in regulating CDK4/6 inhibitor resistance. Single-cell sequencing data from 23 breast cancer patients were analyzed to explore the core molecular mechanisms underlying this resistance. Transcriptomic analysis and Western blotting were conducted to assess the expression of the NEK10/p53/CDKN1A/CDK2 signaling pathway in breast cancers. Data from 1092 patients in TCGA were also incorporated, alongside a prognostic analysis of 25 clinical samples.

**Results:**

ECM-derived biomechanical signals suppressed CDK4/6 inhibitor-induced cell cycle arrest and senescence in breast cancer cells, promoting drug resistance. scRNA-seq and tumor tissue analysis identified NEK10 as a key downregulated kinase associated with resistance. Mechanistically, ECM-induced mechanical forces reduced NEK10 expression via a cytoskeleton-dependent pathway, leading to suppression of the NEK10/p53/CDKN1A axis and activation of CDK2 signaling. NEK10-deficient cells and organoids displayed enhanced resistance to Palbociclib, which was reversed by co-treatment with the CDK2 inhibitor. *In vivo*, combined inhibition of CDK4/6 and CDK2 significantly improved therapeutic efficacy in NEK10-low breast cancer.

**Conclusion:**

This study underscores the critical role of ECM-derived biomechanical forces in regulating CDK4/6 inhibitor resistance in breast cancer and identifies NEK10 as a potential therapeutic target for improving breast cancer treatment.

## Introduction

Breast cancer is the most commonly diagnosed cancer and the leading cause of cancer mortality among women worldwide. The estimated new cases reached 2.3 million in 2022, comprising 11.6% of all cancer cases, and 666 000 cases died of it^[1]^. In recent years, CDK4/6 inhibitors have emerged as a promising targeted therapy, demonstrating significant progress in the treatment of hormone receptor-positive and human epidermal growth factor receptor-2 negative (HR+/HER2−) advanced breast cancer^[2]^. These inhibitors primarily function by inhibiting cyclin-dependent kinases 4 and 6 (CDK4/6), thereby blocking the transition of cells from the G1 phase to the S phase and suppressing tumor cell proliferation^[3]^. While CDK4/6 inhibitors have substantially extended progression-free survival and overall survival, their therapeutic efficacy and tolerability remain challenging. Drug resistance poses a significant hurdle in their clinical application, as patients who initially respond to treatment often develop acquired resistance after several months. This resistance is associated with factors such as RB protein loss, CDK6 gene amplification, and Cyclin E1 overexpression^[4]^. However, the precise mechanisms underlying resistance to CDK4/6 inhibitors in breast cancer patients are still not fully understood.

The extracellular matrix (ECM) is a crucial component of the tumor microenvironment that significantly influences tumor initiation, progression, and metastasis by providing both biophysical and biochemical support^[5]^. Dynamic remodeling of the ECM, including collagen degradation, deposition, and cross-linking, markedly alters the structural and functional characteristics of tumors^[6]^. Recent evidence suggests that the ECM not only regulates cellular behavior through chemical signals but also exerts profound effects on tumor cells through biomechanical forces^[7]^. Mechanical properties of the ECM, such as stiffness, fiber alignment, and architectural changes, interact with cell surface receptors, activating intracellular signaling pathways that promote carcinogenic processes, including epithelial-mesenchymal transition, cell migration, and invasion^[8]^. Additionally, extracellular mechanical forces can propagate long-range mechanical signals through cytoskeletal prestress, influencing chromatin remodeling and gene expression, thereby directly regulating tumor cell biology^[9]^. Studies have shown that increased matrix stiffness is a hallmark of several cancers, including pancreatic and colorectal cancers, and is closely associated with tumor invasiveness and therapeutic resistance^[10]^. ECM components also interact with cell membrane receptors, such as integrins and growth factor receptors, activating signaling pathways (e.g., focal adhesion kinase/Src and protein kinase A/Smad-1) that drive tumor progression^[11,12]^. However, the specific roles of ECM components and mechanical forces vary across tumor types and stages of progression and are not fully understood. Despite advancements in understanding ECM’s role in tumor biology, significant research gaps remain. First, the interactions between ECM components and their specific mechanisms of influence on tumor cell biology are not fully elucidated, with distinct ECM components exerting different effects across various tumor types. Second, current studies on ECM-derived biomechanical forces and their impact on tumor behavior are limited, particularly concerning how these forces contribute to the development of drug resistance in cancer cells. Modulating the mechanical properties of the ECM, such as altering matrix stiffness or fiber alignment, holds potential as a strategy to intervene in tumor progression and could form the basis for developing novel anticancer therapies in the future.

Our previous research demonstrated that ECM-derived biomechanical forces regulate the stemness of breast cancer cells^[13]^. In this study, we further investigated the impact of these biomechanical forces on CDK4/6 inhibitor resistance in HR+, HER2− breast cancer. The results revealed that ECM-derived biomechanical forces promote resistance to CDK4/6 inhibitors in HR+, HER2− breast cancer by suppressing the expression of the key molecule NIMA-related kinase 10 (NEK10). Mechanistically, we showed that these forces influence CDK4/6 inhibitor resistance through the NEK10/p53/CDKN1A/CDK2 signaling pathway. These findings underscore the critical role of biomechanical forces in modulating CDK4/6 inhibitor resistance in breast cancer, offering new insights into the interplay between biomechanics and oncology. Our study also identifies potential biomarkers for the diagnosis and treatment of HR+, HER2− breast cancer. This article adheres to the TITAN Guidelines 2025 (TITAN Working Group, 2025), which govern the transparent and responsible use of AI in scientific publications^[14]^.

**HIGHLIGHTS**

- ECM-derived biomechanical forces promote CDK4/6 inhibitor resistance in HR+, HER2− breast cancer.
- Mechanical cues suppress NEK10 expression via a cytoskeleton-dependent mechanism.
- NEK10 loss activates the p53/CDKN1A/CDK2axis, bypassing CDK4/6 inhibition.
- Low NEK10 expression correlates with poor prognosis and therapeutic resistance.
- Combined CDK4/6 and CDK2 inhibition reverses resistance in NEK10-deficient tumors.

## Materials and methods

### Clinical specimens and TCGA information

Twenty-five human breast tumor tissue samples (HR+/HER2−) were collected from the Sun Yat-sen University Cancer Center. The inclusion criteria for patient selection were as follows: (1) histologically confirmed hormone receptor-positive and HER2−negative breast cancer; (2) availability of complete clinical follow-up data for a minimum of 5 years after surgical treatment; and (3) patients treated with CDK4/6 inhibitors. Patients were excluded if they met any of the following criteria: (1) incomplete clinicopathological data; or (2) receipt of non-standard therapeutic regimens. Patients were stratified into two groups based on their recurrence status during the follow-up period. The non-resistant group (*n* = 15) included patients who remained disease-free for at least 2 years following CDK4/6 inhibitor treatment, whereas the resistant group (*n* = 10) comprised individuals who developed disease progression within 1 years. NEK10 expression in breast cancer samples was assessed by immunohistochemistry (IHC). The staining intensity and proportion of positive tumor cells were semiquantitatively scored to obtain an H-score, and the median H-score value was used as the cutoff to categorize patients into NEK10-high and NEK10-low groups. All patients provided written informed consent prior to sample collection. The study was approved by the Ethics Committee (SL-B2024-317-02) and conducted in accordance with the Declaration of Helsinki. mRNA expression data from 114 normal tissues and 1092 breast tumors were obtained from TCGA and GTEx databases. Differentially expressed genes in high/low NEK10 expressed groups, as well as resistant/non-resistant groups were selected based on the screening criteria of fold change of >2 and *P*-value of <0.05. Survival and transcriptomic data of 382 breast cancer patients from the TCGA database were analyzed using R software.

### Cell line and organoids culture

The human breast cancer cell lines MCF-7, T47D, and ZR-75-1 were obtained from the American Type Culture Collection and cultured in RPMI-1640 medium supplemented with 10% fetal bovine serum at 37 °C in a humidified incubator with 5% CO_2_. Organoids were generated following a previously published protocol^[15]^. Briefly, tissue samples were finely minced and digested with collagenase type A (1.6 U/ml) at 37 °C for 2 hours, followed by centrifugation and filtration. Red blood cells were removed using a lysis buffer, and the isolated single-cell suspension was seeded in 3D Matrigel (5 mg/ml) and plated into 24-well plates. The organoid culture medium, consisting of DMEM (Gibco) with 1x Glutamax, 10 mM Hepes, 1x penicillin/streptomycin, 50 µg/ml Primocin, 1x B27 supplement, 5 mM nicotinamide, 1.25 mM N-acetylcysteine, 250 ng/ml R-spondin 3, 5 nM Heregulin β-1, 100 ng/ml Noggin, 20 ng/ml FGF-10, 5 ng/ml FGF-7, 5 ng/ml EGF, 500 nM A83-01, and 500 nM SB202190, was added to each well and refreshed every 3 days. During the first week, 5 μM Y-27632 was included in the culture. Once organoid colonies reached a size greater than 500 μm, they were digested and passaged. Each organoid culture was maintained for up to 2 months and used for subsequent experiments.

### 3D culture

MCF-7, T47D, and ZR-75-1 cells were seeded in 3D cultures of Matrigel, collagen I, and fibrinogen gels, as previously published^[13]^. The substrate concentration was adjusted to create 3D gels with varying stiffness (Supplemental Digital Content Method and Materials, available at: http://links.lww.com/JS9/G685). A total of 5 × 10^4^ MCF-7, T47D, and ZR-75-1 cells were seeded in 250 μl of 3D gel within a 24-well plate containing 1 ml of culture medium. Cells were cultured in the 3D gels for up to 6 days. The gels were degraded with Dispase (Corning, USA) to isolate the cells. In most experiments, tumor cells were cultured in 3D Matrigel with a stiffness of 45 Pa.

### Single-cell RNA sequencing analysis

We selected tumor cells associated data from 23 breast cancer patients (HR+/HER2−) from the GEO database (GSE158724). Based on their prognosis following CDK4/6 inhibitor treatment, patients were classified into responder and non-responder groups.

#### Single-cell RNA sequencing data processing

Single-cell data matrices were combined using the “merge” function, and sample labels were assigned to each cell. To reduce inter-sample and inter-cell variability, the “NormalizeData” function from the Seurat v4.3.0 R package was used for normalization, employing the global-scaling method “LogNormalize” with a scale factor of 10 000. The data were subsequently log-transformed. The “FindVariableFeature” function, using the “vst” method, identified the top 2000 variable genes for downstream analyses. To prevent overrepresentation of highly expressed genes and improve cross-cell gene expression comparability, the “ScaleData” function was applied to scale gene expression values. Principal component analysis (PCA) was then performed using the “RunPCA” function, with the top 30 principal components (PCs) selected for further analysis. Cells were clustered using the “FindNeighbors” and “FindClusters” functions, and cluster visualization was performed using the “CellDimPlot” function from the SCP v0.4.7R package.

#### Detection of cluster-specific genes

Differentially expressed genes (DEGs) in different cell clusters were identified using the “FindAllMarkers” function from Seurat, applying the Wilcoxon rank-sum test. Cells were also categorized based on NEK10 expression levels, with those above the median defined as NEK10_high and those below as NEK10_low. DEGs between the NEK10_high and NEK10_low groups were evaluated using the Wilcoxon rank-sum test via the “RunDEtest” function from the SCP package.

#### Functional enrichment analysis

Functional enrichment analysis of the DEGs was conducted using the ClusterProfiler package, with all human protein-coding genes serving as the background set. Multiple testing correction was applied using the Benjamini–Hochberg method, with an adjusted *P*-value of <0.05 considered the threshold for significantly enriched pathways. Gene Ontology (GO) and KEGG pathway enrichment analyses were performed to assess the functional roles of the DEGs. GO terms or KEGG pathways with a corrected *P*-value of <0.05, calculated via hypergeometric testing and the Benjamini–Hochberg method, were defined as significantly enriched.

### Animal protocols

Female severe combined immunodeficiency (SCID) mice (6 weeks old) were purchased from Vital River Laboratory Animal Technology Co. (China) and maintained in a specific pathogen-free facility. All animal experiments were conducted in accordance with the guidelines of the Ethics Committee. Organoids derived from NEK10-high and NEK10-low breast cancer patients were encapsulated in 3D Matrigel (45 Pa) and subcutaneously implanted into SCID mice (*n* = 8 per group, 2 × 10^5^ cells per mouse). The mice were treated with PBS, Palbociclib (50 mg/kg, oral, daily), Fadraciclib (20 mg/kg, oral, daily), or Palbociclib combined Fadraciclib for 2 weeks. Treatment started when all tumors reached 100 mm^3^ volume. Tumor volumes and survival data were recorded. This study was reported in accordance with the ARRIVE guidelines (Animals in Research: Reporting *In Vivo* Experiments)^[16]^.

### Statistical analysis

Statistical analysis was performed using SPSS v23.0 or GraphPad Prism v7. Data are presented as mean ± SD for continuous variables, and number (%) for categorical variables, unless otherwise noted. Differences between two groups were assessed using an independent sample *t*-test, while comparisons among multiple groups were performed using one-way ANOVA followed by Tukey’s *post hoc* test. The Kaplan–Meier estimator was employed to evaluate overall patient survival. Each experiment was conducted at least three times independently. A *P*-value of less than 0.05 was considered statistically significant.

## Results

### ECM-derived biomechanical force promotes resistance to CDK4/6 inhibitors in breast cancer

**Figure 1.**
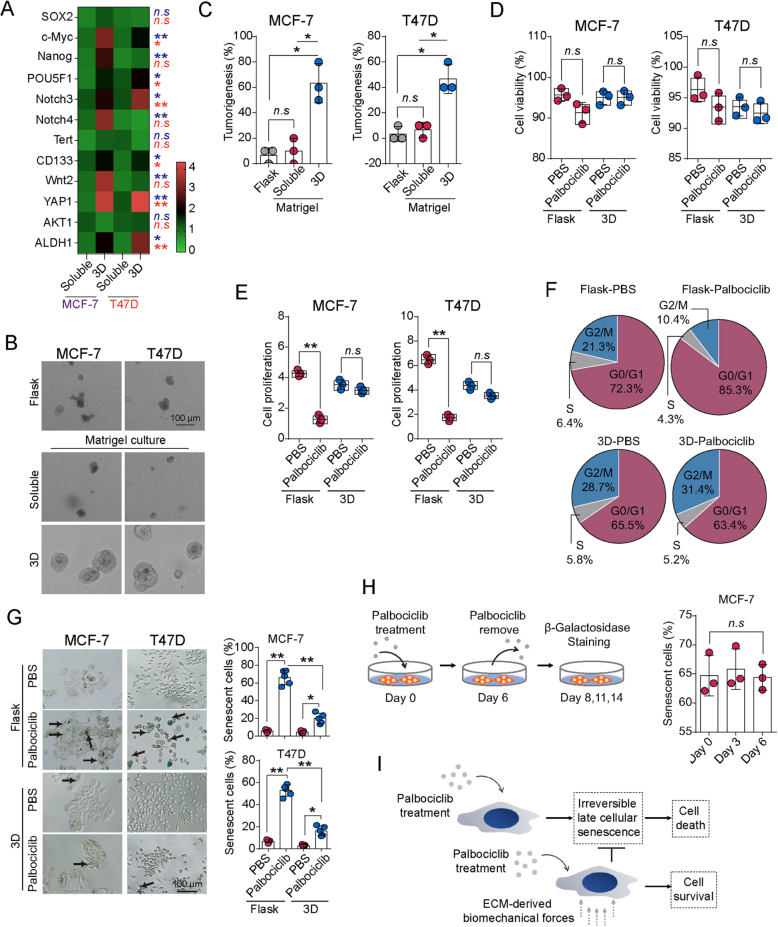
ECM-derived biomechanical force promotes resistance to CDK4/6 Inhibitors. (A) Heatmap showing gene expression levels of SOX2, c-Myc, Nanog, POU5F1, Notch3, Notch4, Tert, CD133, Wnt2, YAP1, AKT1, and ALDH1 in MCF-7 and T47D cells cultured in soluble Matrigel or within 3D Matrigel (45 Pa) for 3 days, as determined by qPCR. Cells cultured in flasks served as the control group. (B) MCF-7 and T47D cells were cultured in soluble Matrigel or within 3D Matrigel (45 Pa) for 3 days, followed by colony formation analysis in agarose soft gel. Scale bar: 100 μm. (C) MCF-7 and T47D cells were cultured in soluble Matrigel or within 3D Matrigel (45 Pa) for 3 days, and *in vivo* tumor formation was assessed (1 × 10^4^ cells per mouse, 20 days). (D ~ G) MCF-7 and T47D cells were cultured in flasks or within 3D Matrigel (45 Pa), treated with Palbociclib (1 μM) for 6 days, and then analyzed for cell apoptosis (D), cell proliferation (E), cell cycle (F), and β-galactosidase staining (G). Scale bar: 100 μm. (H) MCF-7 and T47D cells were cultured in flasks and treated with Palbociclib (1 μM) for 6 days. On day 6, Palbociclib was removed, and cells were cultured in fresh medium. Cells were collected on days 6, 9, and 12 for β-galactosidase staining. (I) Schematic diagram illustrating that ECM-derived biomechanical forces promote breast tumor cell proliferation in a CDK4/6-independent manner, allowing cells to escape CDK4/6 inhibitor-induced senescence. Data are presented as mean ± SD, and analyzed by unpaired *t*-test. *P < 0.05, **P < 0.01, ***P < 0.001.

Our previous research demonstrated that ECM-derived biomechanical forces promote the stemness characteristics of breast cancer cells^[13]^. To further explore the impact of ECM biomechanical signals on breast tumors, specifically regarding CDK4/6 inhibitor resistance, we selected HR+, HER2− breast cancer cell lines MCF-7 and T47D, culturing them in 3D Matrigel (45 Pa) as previously described. Consistent with prior findings, HR+, HER2− breast cancer cells exhibited significantly upregulated expression of stemness genes under matrix-derived biomechanical signals (Fig. 1A), along with enhanced clonogenic capacity *in vitro* (Fig. 1B) and increased tumorigenic potential *in vivo* (Fig. 1C). Next, we examined the effect of 3D ECM-derived biomechanical signals on CDK4/6 inhibitor resistance in HR+, HER2− breast cancer cells. MCF-7, T47D, and ZR-75 cells were cultured in both flask and 3D Matrigel environments and treated with Palbociclib for 6 days. The results showed that 3D culture did not significantly impact apoptosis (Fig. 1D; Supplemental Digital Content S1D, available at: http://links.lww.com/JS9/G685) but markedly reduced the inhibitory effect of Palbociclib on cell proliferation (Fig. 1E; Supplemental Digital Content S1E, available at: http://links.lww.com/JS9/G685), suggesting that biomechanical signals from 3D Matrigel mediate resistance to Palbociclib by influencing the cell cycle rather than directly affecting apoptotic pathways. Further cell cycle analysis revealed that while Palbociclib induced G0/G1 arrest, tumor cells cultured in 3D were not affected by this arrest (Fig. 1F; Supplemental Digital Content S1F, available at: http://links.lww.com/JS9/G685). Given that tumor cells can enter a dormant or senescent state following cell cycle arrest, we performed β-galactosidase staining to assess cellular senescence in MCF-7, T47D, and ZR-75-1 cells treated with Palbociclib. The results revealed significant senescence in cells cultured in flasks under Palbociclib treatment, whereas 3D Matrigel culture significantly suppressed this process (Fig. 1G; Supplemental Digital Content S1G, available at: http://links.lww.com/JS9/G685). These findings indicate that CDK4/6 inhibitors induce cell cycle arrest and senescence in HR+, HER2− breast cancer cells, while biomechanical signals from 3D Matrigel mediate resistance to these inhibitors. To verify that this effect is driven by mechanical rather than chemical signals, we cultured MCF-7 cells in flasks, soluble Matrigel, and 3D Matrigel, and assessed senescence after 6 days of Palbociclib treatment. The results showed that soluble Matrigel could not reverse CDK4/6 inhibitor-induced senescence (Supplemental Digital Content Figure S1A, available at: http://links.lww.com/JS9/G685), suggesting that resistance is primarily mediated by mechanical signals from the 3D matrix. To further investigate the effects of biomechanical signal intensity on CDK4/6 inhibitor resistance, we cultured MCF-7 cells in 3D Matrigel with varying stiffness levels (0, 15, 30, 45, 90, and 450 Pa). We found that biomechanical signals of 30 Pa or 45 Pa mediated resistance, but higher forces (90 Pa and above) did not further enhance the resistance effect (Supplemental Digital Content Figure S1B, available at: http://links.lww.com/JS9/G685). Additionally, various 3D matrices (collagen, fibrin, and Matrigel) all mediated resistance to CDK4/6 inhibitors (Supplemental Digital Content Figure S1C, available at: http://links.lww.com/JS9/G685), further confirming that mechanical signals play a dominant role. Tumor cell senescence can be categorized into reversible early senescence and irreversible late senescence. To determine whether biomechanical signals mediate resistance by reversing early senescence or preventing late senescence, we treated MCF-7 cells cultured on monolayers with Palbociclib for 6 days, then removed the drug and continued culturing the cells, assessing senescence at 0, 3, and 6 days post-Palbociclib removal. The results indicated that the tumor cells were in a state of irreversible late senescence rather than reversible early senescence (Fig. 1H), suggesting that 3D biomechanical signals mediate resistance by preventing the transition to late senescence rather than reversing early senescence (Fig. 1I). Overall, these findings reveal that ECM-derived biomechanical signals inhibit CDK4/6 inhibitor-induced cell cycle arrest and senescence, thereby mediating resistance to CDK4/6 inhibitors in HR+, HER2− breast cancer.

### NEK10 deficiency as a key driver of CDK4/6 inhibitor resistance in breast cancer patients

**Figure 2.**
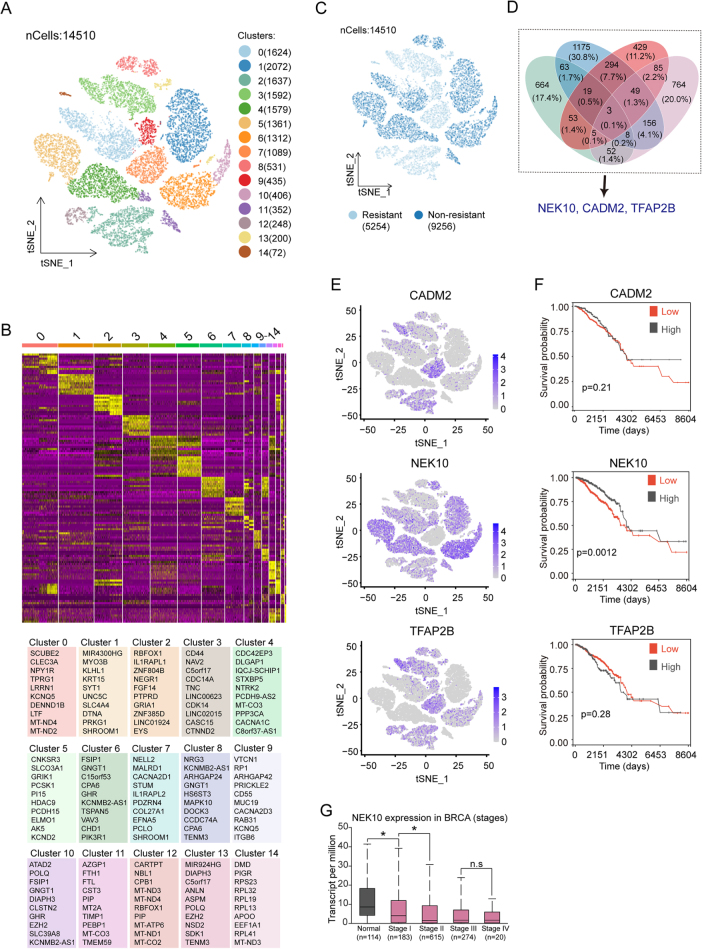
NEK10 deficiency as a key driver of CDK4/6 inhibitor resistance. (A) *t*-SNE plot of 14 tumor cell clusters identified in single-cell RNA sequencing (scRNA-seq) of 23 breast tumor tissues. (B) Heatmap showing the top differentially expressed genes among each tumor cell subtype. (C) *t*-SNE plot depicting the distribution of non-resistant and resistant tumor cells within the 14 identified clusters. (D) The Venn diagram represents the intersection between these DEG sets that were differentially expressed in resistant or non-resistant patients, as well as resistant or non-resistant clusters. (E) *t*-SNE plot illustrating the expression of CADM2, NEK10, and TFAP2B across various tumor cell subtypes. (F) Survival analysis of CADM2, NEK10, and TFAP2 expression in 382 breast cancer patients from the TCGA database. (G) mRNA expression levels of CADM2, NEK10, and TFAP2B in 114 normal tissues and 1092 breast tumors at various stages, sourced from the TCGA database. Kaplan–Meier survival curves were evaluated with log-rank test. All statistical tests were two-tailed. Data are presented as mean ± SD, and analyzed by unpaired *t*-test. *P < 0.05, **P < 0.01, ***P < 0.001.

To investigate the core mechanisms by which extracellular matrix-derived biomechanical signals regulate CDK4/6 inhibitor resistance in breast cancer, we performed single-cell transcriptome sequencing on tumor tissues from 23 HR+, HER2− breast cancer patients from the GEO database. Patients were categorized into CDK4/6-resistant and non-resistant groups based on clinical outcomes following drug treatment. After quality control, 14 510 tumor cells were collected and sequenced. Gene identification was conducted in an unbiased manner, and dimensionality reduction was performed using *t*-distributed Stochastic Neighbor Embedding (*t*-SNE), identifying 15 distinct tumor cell subpopulations (Fig. 2A). Each subpopulation exhibited unique gene expression profiles and signature gene sets (Fig. 2B). Localization analysis revealed that tumor cells from resistant patients were predominantly distributed in subpopulations Cluster 0, 1, 4, 5, 6, 8, and 11 (Fig. 2C). To identify potential resistance-associated genes, we performed two levels of differential gene expression analysis: (1) Between groups – tumor cells from resistant versus non-resistant patients were compared to obtain DEGs associated with clinical resistance; (2) Between enriched clusters – DEGs were also identified between the clusters predominantly enriched by resistant versus non-resistant cells. The Venn diagram represents the intersection between these two DEG sets that were differentially expressed in both comparisons. The three candidate genes shown below the Venn diagram were those consistently identified in both analyses, suggesting that they may play key roles in CDK4/6 inhibitor resistance (Fig. 2D). Notably, CADM2 and TFAP2B were significantly upregulated in resistant tumor cells, while NEK10 was downregulated in the resistant subpopulations (Fig. 2E), suggesting that CDK4/6 inhibitor resistance in HR+, HER2− breast cancer may be associated with the genes CADM2, TFAP2B, and NEK10. To further validate the correlation between CADM2, TFAP2B, and NEK10 with resistance and prognosis in breast cancer, we analyzed prognostic data from 1092 patients in the TCGA and GTEx database. The results showed that low NEK10 expression was significantly associated with poor prognosis, while CADM2 and TFAP2B showed no significant correlation (Fig. 2F; Supplemental Digital Content S2E, available at: http://links.lww.com/JS9/G685). Additionally, compared to normal tissues, NEK10 expression was significantly downregulated in breast cancer tumors, with further decreases observed in advanced stages (Fig. 2G). These findings suggest that NEK10 may be a key molecule influencing CDK4/6 resistance and prognosis in breast cancer.

### NEK10 deficiency enhances CDK4/6 inhibitor resistance both in vitro and in vivo

**Figure 3.**
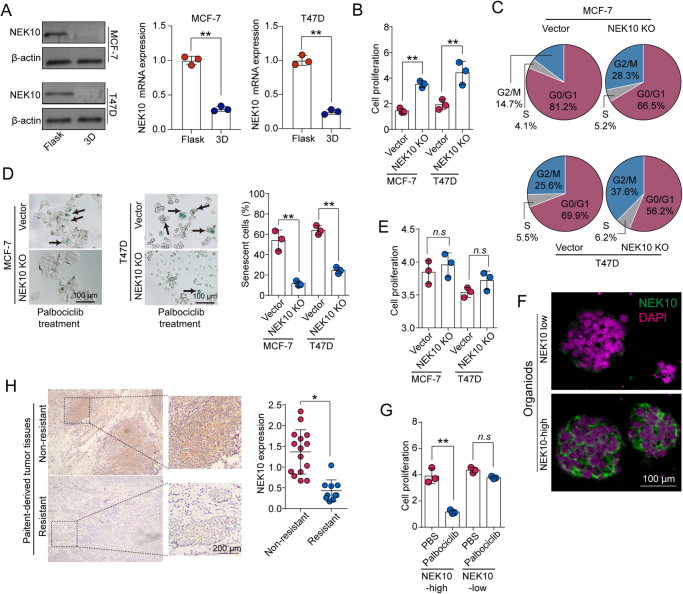
NEK10 deficiency enhances CDK4/6 inhibitor resistance. (A) Western blot and mRNA analysis of NEK10 expression in MCF-7 and T47D cells cultured in flasks and 3D Matrigel. (B–D) Vector or NEK10 KO MCF-7/T47D cells were treated with Palbociclib (1 μM) for 6 days. Cell proliferation (B), cell cycle progression (C), and β-galactosidase staining (D) were analyzed. (E) Vector or NEK10 KO MCF-7/T47D cells were seeded in 3D Matrigel (45 Pa), followed by treatment with Palbociclib (1 μM) for 6 days. Cell proliferation was analyzed. (F) Representative images of organoids derived from breast cancer patients with high and low NEK10 expression. Scale bar: 100 μm. (G) Organoids from patients with high and low NEK10 expression were treated with either PBS or Palbociclib (1 μM) for 6 days. Subsequent cell proliferation analysis was performed. (H) Immunostaining of NEK10 in breast cancer tissues from resistant (*n* = 15) and non-resistant (*n* = 10) patient groups. Scale bar: 200 μm. Data are presented as mean ± SD, and analyzed by unpaired *t*-test. *P < 0.05, **P < 0.01, ***P < 0.001.

To further validate the role of NEK10 in biomechanical signal-mediated CDK4/6 inhibitor resistance, we performed Western blot analysis on MCF-7 and T47D cells cultured in flasks and 3D Matrigel to assess NEK10 expression levels. The results showed a significant decrease in NEK10 expression in MCF-7 and T47D cells cultured in 3D Matrigel (Fig. 3A), suggesting that extracellular matrix-derived biomechanical signals downregulate NEK10 expression in breast cancer cells. To investigate the regulatory role of NEK10 in CDK4/6 inhibitor resistance, we knocked out NEK10 in MCF-7, T47D, and ZR-75-1 cells, treated them with Palbociclib for 6 days, and evaluated cell proliferation (Fig. 3B; Supplemental Digital Content S3A, available at: http://links.lww.com/JS9/G685), cell cycle arrest (Fig. 3C; Supplemental Digital Content S3B, available at: http://links.lww.com/JS9/G685), and cellular senescence (Fig. 3D; Supplemental Digital Content S3C, available at: http://links.lww.com/JS9/G685). These results were further confirmed in NEK10 knockdown MCF-7 and T47D cells by siRNA (Supplemental Digital Content Figure S3D–F, available at: http://links.lww.com/JS9/G685). NEK10 knockout significantly enhanced CDK4/6 inhibitor resistance in these cell lines. We then cultured NEK10-knockout MCF-7, T47D, and ZR-75-1 cells in 3D Matrigel and treated them with Palbociclib. Under 3D culture conditions, NEK10 knockout did not significantly affect CDK4/6 inhibitor resistance, including cell proliferation, cell cycle arrest and cellular senescence(Fig. 3E; Supplemental Digital Content S3G–J, available at: http://links.lww.com/JS9/G685), indicating that biomechanical signals mediate resistance by downregulating NEK10 expression. To further validate this conclusion, we isolated primary tumor tissues with high and low NEK10 expression from HR+, HER2− breast cancer patients and established organoid culture models (Fig. 3F). Organoids with low NEK10 expression exhibited significantly greater CDK4/6 inhibitor resistance compared to those with high NEK10 expression (Fig. 3G). Additionally, we analyzed paraffin-embedded tumor tissue sections from 25 breast cancer patients (HR+/HER2−) before CDK4/6 inhibitor treatment, classifying patients into resistant and non-resistant groups based on clinical follow-up and the clinicopathologic feature, as shown in Supplemental Digital Content Figure S3K, available at: http://links.lww.com/JS9/G685. NEK10 expression was significantly reduced in the tumor tissues of resistant patients (Fig. 3H). Taken together, these results demonstrate that extracellular matrix-derived biomechanical signals promote CDK4/6 inhibitor resistance in breast cancer by downregulating NEK10 expression.

### ECM-derived biomechanical forces suppress NEK10 expression via a cytoskeleton-dependent mechanism

**Figure 4.**
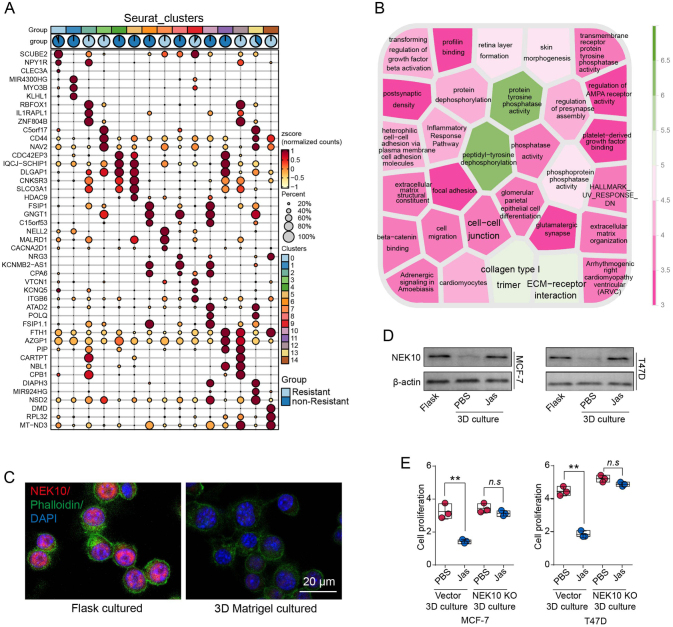
ECM-derived biomechanical forces suppress NEK10 expression via a cytoskeleton-dependent mechanism. (A) Differential gene expression analysis was performed between resistant and non-resistant cell subpopulations from 23 breast cancer patients. (B) Kyoto Encyclopedia of Genes and Genomes (KEGG) enrichment analysis of the differentially expressed genes identified in (A). (C) Phalloidin and NEK10 immunostaining of MCF-7 cells cultured in flasks or 3D Matrigel (45 Pa). (D) Western blot analysis of NEK10 expression in MCF-7 and T47D cells cultured in flasks or 3D Matrigel (45 Pa), treated with either PBS or 20 nM Jasplakinolide. (E) Vector or NEK10 KO MCF-7 and T47D cells cultured in 3D Matrigel were treated with Palbociclib (1 μM) for 6 days, combining 20 nM Jasplakinolide or not. Subsequent cell proliferation analysis was performed. Data are presented as mean ± SD, and analyzed by unpaired *t*-test. *P < 0.05, **P < 0.01, ***P < 0.001.

To explore the mechanism by which ECM-derived biomechanical signals regulate NEK10 expression, we conducted a differential gene expression analysis on tumor cells from 23 breast cancer patients. The patients were categorized into CDK4/6 inhibitor-resistant and non-resistant groups according their progression free survival after CDK4/6 inhibitor treatment. The major DEGs were analyzed among different clusters, as well as resistant and non-resistant groups (Fig. 4A). Gene Ontology and Kyoto Encyclopedia of Genes and Genomes pathway enrichment analyses revealed a significant enrichment of ECM-related signaling pathways, such as ECM-receptor interaction and cell junction pathways (Fig. 4B). Previous studies have demonstrated that ECM-derived mechanical signals can be transmitted via the cytoskeleton, with both ECM-receptor interaction and cell junction processes involving cytoskeletal participation. In our previous study, we had established that ECM-derived biomechanical signals are primarily transduced via integrin receptors and cytoskeletal^[13]^. Consistent with this, in this study we further confirmed that culturing breast cancer cells within ECM-rich 3D matrices significantly upregulated integrin β1 and β3 expression (Supplemental Digital Content Figure S4A, available at: http://links.lww.com/JS9/G685). Based on these findings, we hypothesized that ECM-derived biomechanical signals may regulate NEK10 expression and contribute to CDK4/6 inhibitor resistance through cytoskeletal modulation. To test this hypothesis, we performed immunofluorescence staining for the cytoskeleton (phalloidin staining) and NEK10 in MCF-7 cells cultured on plates and within 3D Matrigel. The results revealed a reduction in cytoskeletal expression and a notable decrease in actin polymerization in MCF-7 cells cultured in 3D Matrigel, alongside decreased NEK10 expression (Fig. 4C). To further investigate the role of the cytoskeleton in NEK10 regulation, we treated MCF-7 cells cultured in 3D Matrigel with an actin stabilizer (Jasplakinolide) and assessed NEK10 expression via Western blot analysis. Although culturing in 3D Matrigel suppressed NEK10 expression, Jasplakinolide suppressed this downregulation (Fig. 4D). These findings suggest that ECM-induced biomechanical signals suppress NEK10 expression through cytoskeletal pathways. Next, we implanted vector control and NEK10-knockout MCF-7 and T47D cells into 3D Matrigel, treated them with Palbociclib, and supplemented the culture medium with either PBS or Jasplakinolide. Cell proliferation was then assessed following treatment. Under 3D culture conditions, Jasplakinolide significantly reversed CDK4/6 inhibitor resistance mediated by biomechanical signals in the vector control group but had no effect on resistance in the NEK10-knockout group (Fig. 4E). Furthermore, we investigated the downstream mechanotransduction pathway. Since integrin-mediated force transmission typically propagates through the cytoskeleton–YAP1 axis, we examined whether this pathway regulates NEK10 expression. Immunofluorescence indicated that YAP1 was remarkably activated in 3D cultured MCF-7 and T47D cells (Supplemental Digital Content Figure S4B, available at: http://links.lww.com/JS9/G685). Additionally, treatment with a YAP1 inhibitor (Vertporfin) markedly suppressed the ECM-induced downregulation of NEK10 under 3D culture conditions (Supplemental Digital Content Figure S4C, available at: http://links.lww.com/JS9/G685). Similarly, integrin inhibitor (GLPG0187) abolished the 3D matrix–mediated downregulation of NEK10 expression (Supplemental Digital Content Figure S4D, available at: http://links.lww.com/JS9/G685). Together, these results indicate that ECM-induced biomechanical signals promote CDK4/6 inhibitor resistance in breast cancer cells by suppressing NEK10 expression via the cytoskeletal associated pathway.

### NEK10 deficiency activates p53/CDKN1A/CDK2 signaling pathways

**Figure 5.**
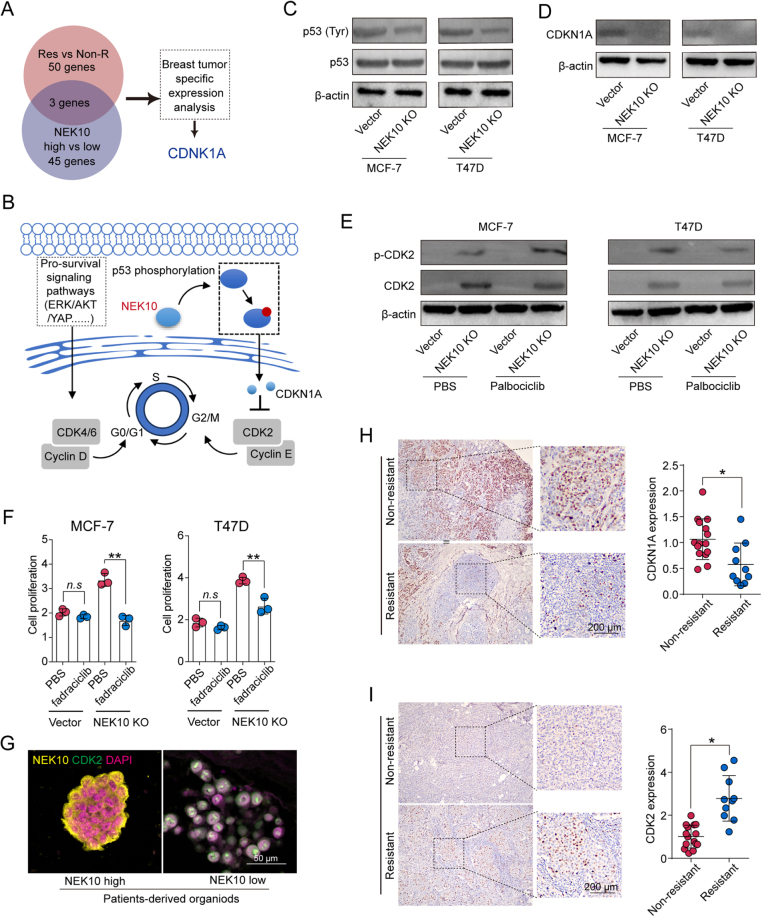
NEK10 deficiency activates p53/CDKN1A/CDK2 signaling pathways. (A) Analysis of the top 50 differentially expressed genes from Figure 4A and the top 45 differentially expressed genes between NEK10 high and low breast cancer patients (*n* = 382) identified CDKN1A as potentially associated with NEK10-mediated breast tumor progression. Breast tumor-specific expression analysis further supported this association. (B) Schematic diagram illustrating that NEK10 promotes the phosphorylation of p53 and upregulation of CDKN1A, leading to the inactivation of CDK2. (C) Western blot analysis of phosphorylated p53 (Tyr) and total p53 in vector and NEK10 KO MCF-7 and T47D cells. (D) Western blot analysis of CDKN1A in vector and NEK10 KO MCF-7 and T47D cells. (E) Western blot analysis of phosphorylated CDK2 (T160), and total CDK2 in vector and NEK10 KO MCF-7 and T47D cells, treated with PBS or Palbociclib (1 μM). (F) vector and NEK10 KO MCF-7 and T47D cells were treated with Palbociclib (1 μM), alone or in combination with Fadraciclib (500 nM). Subsequent cell proliferation analysis was conducted. (G) Immunostaining of CDK2 and NEK10 in organoids derived from NEK10 high and low breast cancer patients. Scale bar: 50 μm. (H) Immunostaining of CDKN1A in breast cancer tissues from non-resistant (*n* = 15) and resistant (*n* = 10) patient groups. Scale bar: 200 μm. (I) Immunostaining of CDK2 in breast cancer tissues from non-resistant (*n* = 15) and resistant (*n* = 10) patient groups. Scale bar: 200 μm. Data are presented as mean ± SD, and analyzed by unpaired *t*-test. *P < 0.05, **P < 0.01, ***P < 0.001.

To further investigate the downstream molecular mechanisms of NEK10 in CDK4/6 inhibitor resistance in breast cancer, we identified a union of differentially expressed genes from both non-resistant and resistant groups, as well as genes specifically expressed in breast cancer patients with high NEK10 levels. We then conducted a breast cancer-specific expression analysis on this gene set. Among the identified genes, only CDKN1A is directly involved in cell cycle regulation, which is highly relevant given that CDK4/6 inhibitors primarily exert their antitumor effects through cell cycle arrest. Therefore, CDKN1A was selected for subsequent investigation as it represents a biologically plausible mediator linking NEK10 to CDK4/6 inhibitor resistance. The analysis suggested that the CDKN1A gene may be closely associated with NEK10-mediated CDK4/6 inhibitor resistance (Fig. 5A). Previous research has demonstrated that NEK10 can promote the phosphorylation of p53, and the activation of phosphorylated p53 induces CDKN1A expression^[17]^. Based on this, we hypothesized that the loss of NEK10 suppresses the p53/CDKN1A signaling axis, subsequently activating downstream CDK2 signaling, allowing the cell cycle to proceed despite CDK4/6 inhibition, thereby contributing to CDK4/6 inhibitor resistance (Fig. 5B). To test this hypothesis, we performed Western blot analysis to examine the expression of p53 and CDKN1A in NEK10-knockout MCF-7 and T47D cells. The results showed that NEK10 deletion significantly reduced the levels of phosphorylated p53 and CDKN1A proteins (Fig. 5C and D). Concurrently, we observed a pronounced activation of the pro-growth CDK2 signaling pathway in the absence of NEK10 (Fig. 5E). Meanwhile, the overexpression of NEK10 significantly suppressed the activation of CDK2 signaling in 3D cultured MCF-7 and T47D cells (Supplemental Digital Content Figure S5A, available at: http://links.lww.com/JS9/G685). These findings suggest that NEK10 loss promotes CDK2 signaling by inhibiting the p53/CDKN1A pathway. We then treated vector control and NEK10-knockout MCF-7 and T47D cells with Palbociclib and supplemented the medium with either PBS or the CDK2 inhibitor Fadraciclib (Fig. 5F). The results demonstrated that the CDK2 inhibitor significantly reversed the CDK4/6 inhibitor resistance caused by NEK10 loss. Consistent with these *in vitro* findings, we examined CDK2 expression in patient-derived organoid models, finding that organoids with NEK10 loss exhibited a significant upregulation of CDK2 expression (Fig. 5G). Additionally, we analyzed tumor tissues from CDK4/6 inhibitor-resistant and non-resistant patients, focusing on the expression of CDKN1A and CDK2. The results revealed a significant downregulation of CDKN1A in resistant patient tumors (Fig. 5H), while CDK2 expression was markedly upregulated (Fig. 5I). Collectively, these findings suggest that NEK10 loss promotes CDK4/6 inhibitor resistance in breast cancer by inhibiting the p53/CDKN1A signaling axis and activating the CDK2 pathway.

### Inhibition of CDK2 enhances the efficacy of CDK4/6 inhibitors in NEK10-deficient breast tumors

**Figure 6.**
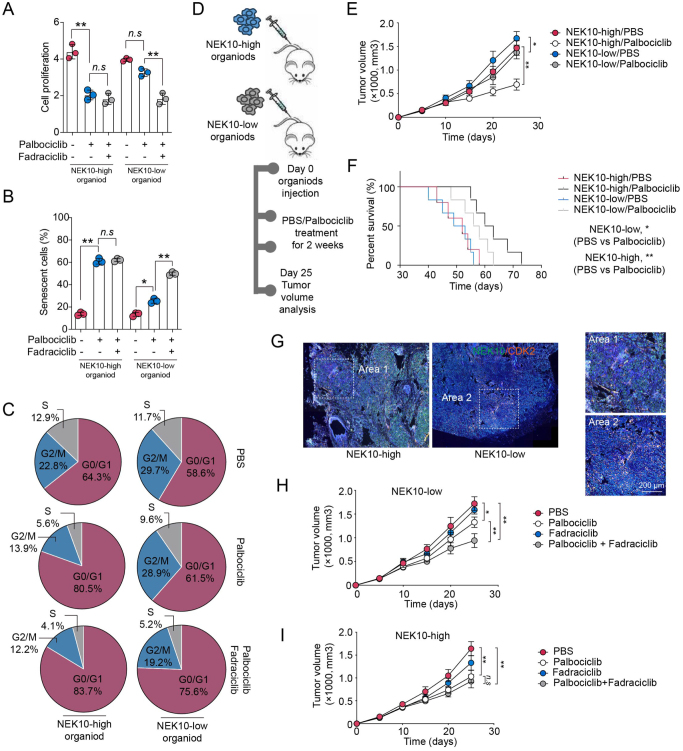
Inhibition of CDK2 enhances the efficacy of CDK4/6 inhibitors in NEK10-deficient breast tumors. (A–C) Organoids derived from NEK10 high and low breast cancer patients were treated with PBS, Palbociclib (1 μM), or Palbociclib combined with Fadraciclib (500 nM) for 6 days. Subsequent analyses included cell proliferation (A), β-galactosidase staining (B), and cell cycle (C). (D–F) Organoids from NEK10 high and low breast cancer patients were subcutaneously implanted into immunodeficient mice. Mice were treated with PBS or Palbociclib (50 mg/kg, oral, daily) for 2 weeks. Tumor volume and mouse survival were analyzed. (G) Immunostaining of NEK10 and CDK2 in tumor tissues from (D). Scale bar: 200 μm. (H and I) Organoids derived from NEK10 high and low breast cancer patients were implanted into immunodeficient mice and treated with a combination of Palbociclib (50 mg/kg, oral, daily) and Fadraciclib (20 mg/kg, oral, daily) for 2 weeks. Tumor volumes were recorded. Data are presented as mean ± SD, and analyzed by unpaired *t*-test. Kaplan–Meier survival curves were evaluated with log-rank test. *P < 0.05, **P < 0.01, ***P < 0.001.

Our previous research demonstrated the critical role of the NEK10/CDK2 signaling pathway in mediating CDK4/6 inhibitor resistance. Based on these findings, we hypothesized that in HR+, HER2− breast cancer patients with NEK10 deficiency, a combined treatment of CDK4/6 inhibitors and CDK2 inhibitors could significantly improve therapeutic outcomes. To test this, we utilized patient-derived organoid models, treating them with Palbociclib alone or in combination with the CDK2 inhibitor Fadraciclib. We then evaluated changes in tumor cell proliferation, senescence, and cell cycle progression. The organoids were categorized into NEK10 high-expression and NEK10 low-expression groups. In the NEK10 high-expression group, Palbociclib effectively inhibited tumor growth, and the addition of Fadraciclib did not result in a significant difference compared to Palbociclib monotherapy. However, in the NEK10 low-expression group, Palbociclib monotherapy exhibited a limited inhibitory effect, while the combination with Fadraciclib significantly enhanced tumor suppression (Fig. 6A–C). These findings suggest that CDK2 inhibition can effectively reverse NEK10-mediated resistance to CDK4/6 inhibitors. To further validate these results, we transplanted NEK10 high-expression and NEK10 low-expression organoids into immunodeficient mice to establish tumor-bearing models, followed by Palbociclib treatment. In the NEK10 high-expression group, Palbociclib effectively inhibited tumor growth in mice. Conversely, in the NEK10 low-expression group, Palbociclib had fewer impact on tumor growth and mouse survival, compared to NEK10 high-expression group (Fig. 6D–F). Additionally, we observed significant activation of CDK2 signaling in the NEK10 low-expression tumor tissues (Fig. 6G). We then treated the tumor-bearing mice with a combination of Palbociclib and Fadraciclib. In order to assess the drug toxicity, we evaluated the weight, alanine transaminase (ALT) and creatinine (CRE) level of mice treated with PBS, Palbociclib, and Palbociclib combined Fadraciclib (Supplemental Digital Content Figure S6A–C, available at: http://links.lww.com/JS9/G685). It was shown that combined treatment had no significant adverse effects on body weight, liver, or kidney function, indicating good short-term tolerability. The results showed that in the NEK10 low-expression group, Fadraciclib significantly enhanced the tumor-suppressive effect of Palbociclib (Fig. 6H), while in the NEK10 high-expression group, Fadraciclib had little impact on Palbociclib’s inhibitory effect (Fig. 6I). These findings suggest that NEK10 could serve as a potential biomarker for predicting response to CDK4/6 inhibitor therapy, and that combined inhibition of CDK2 and CDK4/6 may represent an effective therapeutic strategy for breast cancer patients with NEK10 deficiency.

## Discussion

The ECM within the tumor microenvironment plays a pivotal and dynamic role in tumor initiation and progression. Tumor cell-driven ECM remodeling facilitates irreversible protein cross-linking and proteolysis, modulating growth signals in tumor and stromal cells and influencing chemical and mechanical signaling within the microenvironment^[18]^. Our previous research demonstrated that ECM-derived biomechanical forces regulate stemness pathways in breast cancer cells, thereby impacting cancer initiation and progression^[13]^. Consistently, this study demonstrated that stemness genes are upregulated in the 3D Matrigel cultured breast cancer cells. However, in our results, only a subset of stemness-associated genes (e.g., Nanog, SOX2, and POU5F1) was significantly upregulated under 3D biomechanical culture conditions, while others such as Notch4 and Tert did not show marked increases. This indicates that ECM-derived biomechanical forces selectively regulate stemness-related pathways rather than uniformly enhancing all stemness genes. Such selective regulation suggests a nuanced and context-dependent modulation of tumor stemness by biomechanical cues, which may contribute to the heterogeneity of cancer stem cell phenotypes in breast cancer. In this study, we further demonstrate that ECM-derived biomechanical forces suppress NEK10 expression in HR+, HER2− breast cancer cells via cytoskeletal modulation, contributing to CDK4/6 inhibitor resistance. To our knowledge, this study is the first to reveal the role of ECM biomechanical forces in regulating CDK4/6 inhibitor resistance in HR+, HER2− breast cancer, underscoring the critical role of NEK10 in this process.

Tumor cells remodel the ECM, altering its stiffness and architecture, which activates intracellular mechanotransduction signals, influences the tumor microenvironment, and supports the maintenance and proliferation of cancer stem cells (CSCs)^[19]^. Studies have shown that ECM stiffness activates effectors such as Rho/ROCK and YAP/TAZ via integrin and focal adhesion kinase (FAK) signaling pathways, regulating cytoskeletal tension and nuclear transcription, thereby enhancing tumor cell stemness and invasiveness^[7,20–23]^ . Increased ECM stiffness has also been linked to resistance to various anticancer drugs, including chemotherapy agents^[24]^, and more recently, to resistance against CDK4/6 inhibitors^[25,26]^. While CDK4/6 inhibitors effectively target cell cycle regulation in hormone receptor-positive breast cancer, resistance remains a significant clinical challenge, often associated with ECM remodeling. For instance, collagen in the ECM promotes tumor cell self-renewal and activates alternative survival pathways such as PI3K/AKT/mTOR^[27]^ and EGFR through the integrin-FAK-YAP/TAZ axis, enabling tumor cells to evade drug effects^[28]^. Similarly, laminin enhances the expression of stemness genes (e.g., OCT4, SOX2, and NANOG) through activation of α6β1 integrin and TAZ pathways, further contributing to resistance^[29,30]^. While existing research primarily focuses on the impact of ECM chemical signals on CDK4/6 inhibitor resistance, there is theoretical speculation that ECM mechanical signals might also influence resistance by modulating intracellular stress and chromatin states, thereby affecting stemness gene expression. However, the specific role of these mechanical signals in CDK4/6 inhibitor resistance has not been definitively established. Although prior studies have convincingly linked ECM stiffening and fibrotic remodeling to acquired resistance, they typically emphasize phenotypic associations and canonical mechano-transduction pathways (e.g., integrin/FAK, YAP/TAZ) or altered drug perfusion as explanatory mechanisms. In contrast, our work identifies NEK10 as a specific molecular mediator that connects extracellular matrix cues to intracellular p53/CDKN1A/CDK2 signaling and thereby to resistance to CDK4/6 inhibitors. Thus, ECM remodeling may drive resistance through multiple, potentially parallel mechanisms, and NEK10 represents a druggable nodal point translating ECM changes into cell-cycle reprogramming relevant to CDK4/6 inhibitor efficacy. In this study, we utilized various *in vitro* 3D ECM culture models to demonstrate that biomechanical signals in a 3D environment significantly enhance breast cancer cell resistance to CDK4/6 inhibitors. This research initially highlights the critical role of ECM-derived biomechanical signals in modulating CDK4/6 inhibitor resistance in breast cancer. However, tumor tissues are highly heterogeneous, and even within a small region, mechanical cues such as ECM stiffness, solid stress, or interstitial fluid pressure can vary dramatically. Thus, there is a limitation in investigating how mechanosensory cues modulate NEK10-dependent resistance mechanisms *in vivo* tissue biomechanics.

NEK10 is a member of the NIMA-related kinase (NEK) family, primarily involved in regulating the cell cycle, DNA damage repair, and mitosis^[31]^. Recent studies have increasingly highlighted the role of NEK10 in tumorigenesis and cancer progression, particularly its potential mechanisms in cancer drug resistance^[17]^. NEK10 is frequently overexpressed in various cancers, including breast, lung, and melanoma, where it regulates cell proliferation and maintains cellular homeostasis, suggesting a significant role in cancer resistance^[32,33]^. Abnormal expression of NEK10 has been strongly linked to resistance against anticancer drugs, which primarily inhibit the G1 phase of the cell cycle. Evidence suggests that NEK10 expression is tightly associated with the activation of β-catenin signaling, a critical mechanism in the development of drug resistance^[34]^. NEK10 also regulates cell cycle-related genes, such as Cyclin D1 and CDK2^[35,36]^, potentially enabling tumor cells to bypass the inhibitory effects of CDK4/6 inhibitors and continue proliferating. Unlike previous studies describing NEK10 as a direct regulator of cell-cycle genes such as Cyclin D1 or CDK2, our findings reveal a distinct mechanism in which NEK10 modulates CDK2 activity through the p53/CDKN1A (p21) axis. This indirect regulation establishes a mechanistic connection between NEK10 signaling and CDK4/6 inhibitor resistance, highlighting a novel aspect of NEK10 function in breast cancer. Additionally, NEK10 is involved in regulating autophagy, a self-protective process that maintains cellular homeostasis and survival by clearing damaged organelles and proteins^[37]^. NEK10 influences autophagic activity through the mTOR signaling pathway, which is associated with resistance to various anticancer drugs, including CDK4/6 inhibitors^[38]^. Moreover, NEK10 plays a role in cytoskeletal remodeling and mechanotransduction, impacting cell adhesion and migration and highlighting its potential involvement in biomechanical signaling regulation^[39,40]^. In this study, we found that the loss of NEK10 is strongly associated with resistance to CDK4/6 inhibitors in HR+, HER2− breast cancer. We further demonstrated that biomechanical signals from the ECM suppress NEK10 expression via cytoskeletal pathways, contributing to drug resistance. These findings underscore the critical role of NEK10 in mediating CDK4/6 inhibitor resistance in breast cancer.

CDK1/2/3 are key members of the Cyclin-Dependent Kinases family, which play critical roles in regulating the cell cycle, DNA repair, and apoptosis, contributing significantly to tumorigenesis and cancer progression^[41]^. Abnormal activation of CDK1/2/3 is closely linked to increased proliferation, invasion, and drug resistance in various cancers, positioning CDK1/2/3 inhibitors as promising therapeutic agents in cancer treatment. Recent advancements have accelerated the development of these inhibitors. For instance, the CDK2 inhibitor effectively suppresses tumor cell proliferation and induces apoptosis by blocking the G2/M phase of the cell cycle^[42]^. Additionally, CDK1 inhibitors enhance the sensitivity of tumor cells to conventional chemotherapy agents, such as paclitaxel and cisplatin, by downregulating DNA repair genes and impairing DNA damage repair, thereby improving drug efficacy^[43]^. CDK2 inhibitors can synergize with other targeted agents, such as PARP inhibitors, to collectively inhibit DNA repair capabilities in cancer cells, demonstrating significant anticancer synergy^[44]^. In our study, we discovered that breast cancer cells with low NEK10 expression activate CDK2 pro-growth signals through the p53/CDKN1A pathway, thereby resisting CDK4/6 inhibitor-mediated cell cycle arrest. Mouse experiments further showed that CDK2 inhibitors significantly enhance the tumor-suppressive effects of CDK4/6 inhibitors in NEK10-low-expressing breast tumors. However, in this study, we limited the duration of the *in vivo* experiment based on ethical considerations and institutional animal welfare guidelines. Tumor-bearing mice were euthanized once tumors reached the predetermined maximum allowable volume. Thus, it was lack of survival curve analyses in CDK4/6 inhibitors combined with CDK2 inhibitors. These findings offer new insights into personalized treatment strategies, suggesting that selecting appropriate CDK inhibitors based on a patient’s molecular profile could optimize therapeutic outcomes.

Despite the comprehensive analyses, several limitations should be noted. First, the clinical application and validation of the inhibitors used in this study remain limited, and their translational potential requires further investigation. Second, the number of clinical specimens, particularly those representing resistant and non-resistant cases, was relatively small, potentially affecting the statistical robustness. Third, the precise mechanisms by which biomechanical signals regulate NEK10 expression through the cytoskeleton remain unclear. Fourth, the role of biomechanical cues in breast cancer therapy and their interaction with cell-cycle–related kinases remain poorly understood and warrant future study. Finally, the predictive and causal relevance of NEK10 signaling in mediating CDK4/6 inhibitor resistance should be further validated using prospective patient cohorts. Moreover, the clinical application of NEK10 as a diagnostic and therapeutic target, as well as the effectiveness of CDK2 inhibitors in breast cancer patients, require further clinical validation. The detection of biomechanical signals in tumor tissues also presents technical challenges.

## Conclusion

Our findings demonstrate that ECM-derived mechanical forces can promote CDK4/6 inhibitor resistance in HR+, HER2− breast cancer through NEK10/p53/CDKN1A/CDK2 signaling, suggesting the potential diagnostic value of NEK10 in breast cancer.

## Data Availability

Data generated and analyzed in this study are included in the manuscript and supplementary files. Additional information is available from the corresponding author on reasonable request.
